# Roles of three Es-Caspases during spermatogenesis and Cadmium-induced apoptosis in *Eriocheir sinensis*

**DOI:** 10.18632/aging.101454

**Published:** 2018-05-24

**Authors:** Ya-Ru Xu, Wan-Xi Yang

**Affiliations:** 1 The Sperm Laboratory, College of Life Sciences, Zhejiang University, Hangzhou 310058, China

**Keywords:** caspase, p53, apoptosis, spermatogenesis, Cadmium

## Abstract

Functions of Caspases remain obscure in Crustacea. We studied the existence and participations of apoptosis-related factors in *Eriocheir sinensis* testis. Three Es-Caspases (Es-Caspase 3/ 7/ 8) in *E. sinensis* were cloned and characterized. We observed that three *es-caspases* mRNA had specific expression patterns during spermiogenesis, with weak signal around the nucleus and invaginated acrosomal vesicle in early-stage spermatids, became stronger in middle-stage, finally focused on the acrosomal tube and nucleus in mature sperm. We then investigated the immunostaining intensity and positional alterations of Es-Caspase 3, Es-Caspase 8 and p53 during spermatogenesis, which were correlated with the differential tendencies of cells to undergo apoptosis and specific organelles shaping processes. After apoptotic induction by Cadmium, Es-Caspase 8 increased gradually, while Es-Caspase 3 increased firstly and then decreased, Es-p53 initially decreased and then increased. These results implies that Es-Caspase 3/ Es-Caspase 8/ p53 may play roles in Cadmium-induced apoptosis during spermatogenesis, and Caspase 8-Caspase 3-p53 pathway may interact with extrinsic or intrinsic pathways to regulate the destiny of sperm cells. Our study revealed the indispensable roles of Caspases during spermatogenesis and the possible molecular interactions in response to the Cadmium-induced apoptosis in *E. sinensis,* which filled the gap of apoptotic mechanisms of crustacean.

## Introduction

Apoptotic programmed cell death (PCD) is a primary type of cell death, initiated and executed jointly by a series of apoptosis-related factors, like a class of cysteine-dependent aspartate-directed proteases (Caspases) [1,2]. Lots of biochemical and morphological characteristics typically occur during apoptosis like chromatin and nuclear condensation, DNA cleavage, organelles destruction, membrane blebbing, apoptotic bodies formation and so on [3–5]. The above apoptosis-related events are caused by extrinsic apoptotic pathway and intrinsic apoptotic pathway. Specific stimuli induce the binding of death receptors (like tumor necrosis factor receptor-TNFR) on the cell membrane with their corresponding ligands (like tumor necrosis factor-TNF) outside the cell, which will trigger the extrinsic Caspase-cascade [6]. Bcl-2 family members or Caspase 2 may facilitate the releasing of Cytochrome c from mitochondrial intermembrane space, and result in cell death via mitochondrial pathway [7,8]. Many factors are responsible for apoptosis, including domestic and external factors, inherited and genetic factors, etc. For example, drugs or metal elements treatment, growth factors shortage, radiation induction, oncogene overexpression or mutation convergence, can all cause cell death [9–11]. The apoptotic cysteine proteases had been found to play indispensable roles in the process of apoptosis since the first discovery in 1985 [12]. They are divided into initiator Caspases and effector Caspases according to the specific protein domains and their functions. Caspase 8 and/or Caspase 2 are classified into the initiator Caspase family as the death effector domains (DED) or caspase-recruitment domains (CARD) in the N-teminus [13]. DED or CARD could mediate signals dimerization and/or upstream adapter molecules recognition. Caspase 3 is the canonical effector Caspase, which could react to both death-inducing signaling complex (DISC)-mediated extrinsic pathway and Bcl-2 family-Caspase 9-mediated intrinsic pathway. Substrates of Caspases affect all aspects of cell life, such as transcriptional factors, cytoskeletal proteins, kinases, and so on [13,14].

Although the apoptotic signal network in mammals are getting clearer, it is still vague in invertebrates such as crustacean. In recent years, various cell apoptotic molecules were isolated and their functions were explored from different crustacean species. The presence and functions of Caspase family members have been studied in *Penaeus merguiensis* [15,16], the black tiger shrimp *Penaeus monodon* [17,18], the freshwater prawn *Macrobrachium rosenbergii* [19], the white shrimp *Litopenaeus vannamei* [20], *Portunus trituberculatus* [21], and so on. Similar to mammal, the main functions of Caspases are related to cell apoptosis. The enhanced caspase-3 activity by *Vibrio alginolyticus* is found to induce haemocytes apoptosis and DNA fragmentation in *Litopenaeus vannamei* [20]. What's more, the apoptotic process involved Caspase 3 may enfeeble the vigor of WSSV-challenged shrimp [22]. The eukaryotic expression of crustacean Caspases in Sf-9 cells exhibited their pro-apoptotic activity, such as cell membrane blebbing, pyknotic nuclei, and so on [16,18]. On the other hand, Caspases play significant roles in immune response. With the infection of infectious hypodermal and haematopoietic necrosis virus (IHHNV), the enzyme activity of Casp3c in *Macrobrachium rosenbergii* increased, which suggested its defensive involvement in body immunity [19]. Beyond that, other key mammalian homologous proteins involved in cell fate determination were also discovered. Inhibitor of apoptosis protein (IAP), acting as the anti-apoptosis factor, was identified from *Penaeus monodon* and *Litopenaeus vannamei* [23,24]. The mechanism of action by Pm-IAP was found to interact with high temperature requirement A2 (HtrA2) and Caspases [25]. Among these, the Chinese mitten crab *Eriocheir sinensis* is an important freshwater aquatic economic species, and studying on cell apoptosis mechanism in *E. sinensis* may push the aquaculture performance of this species forward. The apoptosis-related elements a disintegrin and metalloprotease 10 (ES-ADAM10) and ES-ADAM17 were tested during spermatogenesis in Chinese mitten crab, their up-regulation reacted to etoposide treatment suggesting its influence on germ cell apoptosis [26]. As the "Guardian of the Genome'', the sequence and expression mode of p53 during *E. sinensis* spermiogenesis were confirmed by our lab, and its upstream regulators and the downstream targets still need further investigation [27]. In addition was another key factor, which may carry out an extrinsic apoptotic pathway, TNF-α factor characterized in 2014 [28]. Above all, there are still several other unknown mechanisms in *E. sinensis* to be studied and detailed.

The industrial wastewater containing Cadmium is discharged casually and cause long-term adverse effects to the aquatic organisms, like crustaceans. The differential enzymes expression profiles in *E. sinensis* were investigated in acute and chronic Cadmium-exposed conditions [29]. Results revealed the physiological and ultrastructural damage in response to Cadmium exposure were attributed to increased oxidative stress and the broken sulfhydryl homeostasis [29]. What's more, the antioxidant ability elevated and the metabolizability dropped. Further, studies showed that Cadmium exposure cause problems to the reproductive system and reduce the quality and number of sperms, even resulting in infertility [30,31]. In the freshwater crab *Sinopotamon henanense*, TEM results showed that the sperm membrane, nuclear envelope, chromatin and acrosomal membrane were changed and testis showed severe histopathological lesions after Cadmium-induction [30,31]. The potential mechanisms were as follows. Firstly, Cadmium increased the production of reactive oxygen species (ROS) and changed the antioxidant defense systems, reflecting in the elevated activity of MDA and the descending activities of SOD, GPx and CAT. Secondly, Cadmium activated endonuclease and caused the DNA fragmentation. In mammals, p53 is an active participant in DNA repair and DNA damage response pathways [32]. In *E. sinensis* testis, p53 was also found to mediate spontaneous germ cell apoptosis to eliminate defective germ cells [33]. What's more, the mitochondria-dependent apoptotic pathway involving caspase 3 and caspase 9 was initiated. In *E. sinensis* hepatopancreas, similar toxic effects of Cadmium on the crab were explored [34]. Similarly, the production of ROS and the involvement of caspase-dependent apoptosis could illuminate the origins of tissue damage.

The quantity and high-quality mature sperm depend on the precise regulation of spermatogenesis. Spermatogenesis in *E. sinensis* is a classical research model for crab sperm on account of its two distinguishing features: the gradual formation of the invaginated acrosome from proacrosomal granules and the formation of a cupped nucleus [27,35]. Mature sperm undergo dramatic morphological changes as they differentiate from round cells into highly polarized, cupped, anurans sperm capable of participating in fertilization. The bulk volume of the whole cell and nucleus in spermatogonia are larger than those in the spermatocytes. As the early spermatids, stage I spermatids possess a round nucleus, and the proacrosomal granules in the cytoplasm mature to gather together [35,36]. During the formation of the proacrosomal vesicle, the nucleus of stage II spermatids begin to sink and present a shape of half-moon. In the stage III spermatids, the structure of the acrosomal vesicle (AV) is gradually developed. The mature acrosome is composed of acrosome cap (AC), acrosome tube (AT), fibrous layer (FL), middle layer (ML), and lamellar structure (LS) in mature sperm [22,36]. The nucleus in mature sperm looks like a cup. Given the above, the apparatuses that emerge during the spermatogenesis of *E. sinensis* have unique characters in quality, quantity and function, which are quite different with that in mammals. What's more, as a seasonally breeding and migration species, the molecular mechanisms underlying Chinese mitten crab reproduction will be more complicated to adapt to the changes of climate and environments [37]. Therefore, the spermatogenesis of Chinese mitten crab is a suitable biological model for the investigation of crustacean reproduction.

For Southeast Asia region, Chinese mitten crab is an economically important species with tastiness and high nutrition [38,39]. Nevertheless, for Europe and North America region, Chinese mitten crab is a nonindigenous species which is posing threats to native species and ecosystems [40,41]. Given the above facts and contradictions, the advancing of our understanding on the mechanism of organogenesis has taken an epic importance, especially the mechanism of male germ development. Based on the indispensable roles of Caspases in mammals, the mechanism of cell fate determination during the aging and developmental process of spermatogenesis involving Caspases in the crustacean *E. sinensis* deserves investigation for comparative reasons. In addition there might be new insights to be gained from this animal model for the understanding of human reproduction and aging. Our knowledge of the biological implications and mechanistic aspects of male germ cell death in *E. sinensis* testis in general is incomplete. In the present study, three apoptotic factors (Es-Caspase 3/ Es-Caspase 7/ Es-Caspase 8) were isolated and cloned from the testis of *E. sinensis*. Then the mRNA and protein levels of three Es-Caspases in different tissues were examined by semi-quantitative RT PCR and western blotting respectively. In order to unveil the possible relationship between the expression pattern of Caspases, p53 and various developmental spermatogenic cell stages, we conducted *in-situ* hybridization and immunofluorescence in the present study. In addition, Cadmium treatment led to the expression changes of three apoptotic factors, which implied the active involvement of Es-Caspase 3/ Es-Caspase 8/ p53 in apoptosis. Our study aims to highlights the possibility that Es-Caspases and p53 are involved at all stages of testis development, to support the entire spermatogenesis proceeding effectively and to control the quality and death of germ cells. This discovery might help us to understand better the molecular events involved in the apoptosis of crustacean or invertebrates in general.

## RESULTS

### Full-length cloning and characterization of *es-caspase 3*, *es-caspase 7* and *es-caspase 8*

The full-length sequences of three es-caspases were submitted to GenBank successfully, and the GenBank accession numbers were as follows: *es-caspase 3* (MH183147), *es-caspase 7* (KT161946.1), *es-caspase 8* (KT161947.1). The open reading frame (ORF) of *es-caspase 3* is 1344bp, encoding 447 amino acids (aa) (Figure S1 A). The predicted molecular weight (Mw) of Es-Caspase 3 is 49 kDa and the isoelectric point (pI) is 6.00. The ORF of *es-caspase 7* is 1062bp, encoding 353aa (Figure S1 B). The predicted Mw is 40 kDa and the pI is 6.32. The ORF of es-*caspase 8* is 1572bp, encoding 523aa (Figure S1 C). The predicted Mw is 59 kDa and the predicted pI is 5.25. Through PROSITE prediction of secondary structure, each Caspase could be divided into three parts (Figure S2). Es-Caspase 3 has the pro-domain (1-145aa), p20 domain which contains putative active sites (207-330) and p10 domain (359-418aa) (Figure S2 A1). Es-Caspase 7 owns a short pro-domain (1-90aa), p20 domain (102-229aa), and p10 domain (235-349aa) (Figure S2 B1). Es-Caspase 8 is made of a DED domain (25-110aa), p20 domain (235-370aa), and p10 domain (375-468aa) (Figure S2 C1). Three Caspases may share identical conservative sequences and similar functional sites by comparison with other known sequences and domains. Es-Caspase 3 shares 15.7% overall sequence similarity with that of *Culex quinquefasciatus*, 15.3% with *Drosophila melanogaster*, 14.6% with *Macrobrachium rosenbergii*, and 12.9% with *Litopenaeus vannamei* (Figure S2 A2). The enzyme active sites NFARG share high similarity with other sequences. The sequence similarity between Es-Caspase 7 and that of *Aedes albopictus*, *Mus musculus*, *Drosophila melanogaster*, *Danio rerio*, *Bactrocera dorsalis* is 32.1%, 23.9%, 24.6%, 27%, 24.8%, respectively (Figure S2 B2). The conserved cysteine active site is located in QACRG. The sequence similarity of Es-Caspase 8 to that of *Mus musculus, Drosophila melanogaster, Crassostrea hongkongensis, Tribolium castaneum* was 17.3%, 17.2%, 16.8%, 16.5%, respectively (Figure S2 C2), and the putative active site was QACRG. The phylogenetic trees construction depicted the phylogeny of each Caspase as follows. Es-Caspase 3 bore closer phylogenetic relationship with crustaceans like *Marsupenaeus japonicus* and *Macrobrachium nipponense*, Es-Caspase 7 is evolutionarily conserved with *Drosophila melanogaster*, and Es-Caspase 8 grouped more closely to *Daphnia magna* (Figure S2 A3-C3). The 3D structural model pointed out the pocket-shaped Caspase cysteine active sites inside the protein (Figure S2 A4-C4).

### The analysis in transcriptional level of three *es-caspases* in different tissues

**Figure 1:**
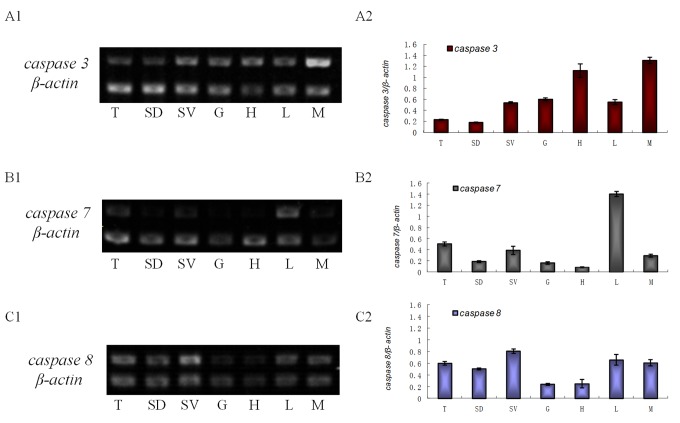
**The transcriptional level of *es-caspase 3*/ *es-caspase 7*/ *es-caspase 8* in different tissues.** Seven tissues were dissected from *E. sinensis*: testis (T), seminiferous duct (SD), seminal vesicle (SV), gill (G), heart (H), hepatopancreas (L) and muscle (M). Histograms in the right were constructed from the agarose gel data in the left. (**A1**-**A2**) The semi-quantitative RT-PCR results of *es-caspase 3*. Higher *es-caspase 3* was expressed in M and H. (**B1**-**B2**) The semi-quantitative RT-PCR results of *es-caspase 7*. Higher *es-caspase 7* was expressed in L and T. (**C1**-**C2**) The semi-quantitative RT-PCR results of *es-caspase 8*. The reproductive system (T, SD and SV) showed higher expression of *es-caspase 8*. β-actin was used as the control. Data are the means of three independent experiments.

**Figure 2:**
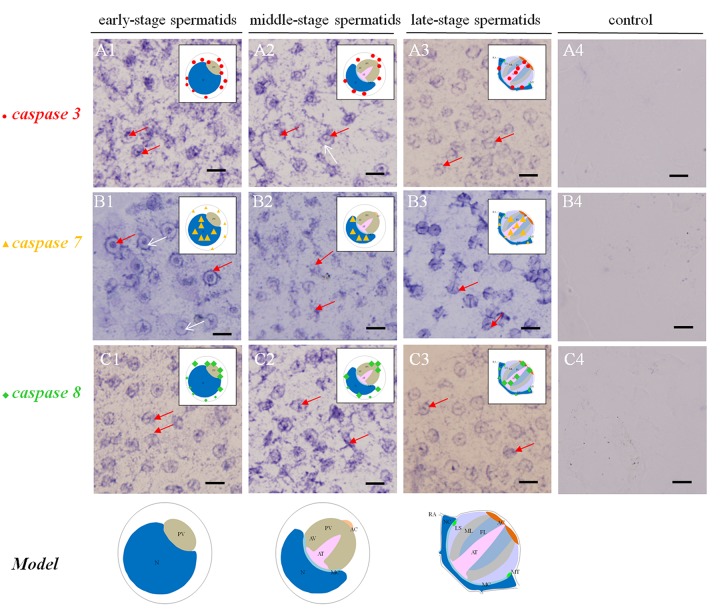
**Temporal and spatial orientation of *es-caspase3*/ *es-caspase7*/ *es-caspase8* during spermiogenesis in *E. sinensis*.** The blue signals in each group were performed by ISH. N: nucleus, PG: proacrosomal granule, PV: proacrosomal vesicle, AT: acrosome tube, AC: acrosome cap, AV: acrosome vesicle, FL: fibrous layer, ML: middle layer, LS: lamellar structure, MC: membrane complex, RA: radical arm, NC: nuclear cap, MT: mitochondria. (**A1**-**A3**) The expression pattern of *es-caspase 3* at various testis stages. In the early spermatids, *es-caspase 3* was distributed in one pole of the cytoplasm and the inner edge of plasma membrane. Signals was decreased in middle-spermatids, and *es-caspase 3* was distributed in the AT and AC finally. (**B1**-**B3**) The expression pattern of *es-caspase 7* during spermiogenesis. Signals were discovered in the nucleus throughout the process. In the mature spermatids, *es-caspase 7* was expressed in the AT, FL, ML and nucleus. (**C1**-**C3**) The distribution of *es-caspase 8* in spermatids. The variation tendency was similar with *es-caspase 3.* The typical spermatid model was in the upper right corner of each panel. (**A4**-**B4-C4**) The control group. Bars=5 um.

Several tissues were selected to do the semi-quantitative RT-PCR: testis, seminiferous duct, seminal vesicle, gill, heart, hepatopancreas and muscle. *es-caspase 3* mRNA had higher expression level in heart, muscle, seminal vesicle, and lower expression level in testis (Figure 1 A1-2). *es-caspase 7* mRNA was highly-expressed in hepatopancreas, testis, and seminal vesicle (Figure 1 B1-2). By comparison, all parts of the reproductive system showed abundant *es-caspase 8* expression, and the level was higher than both *es-caspase 3* and *es-caspase 7* (Figure 1 C-2).

### Changes in *es-caspase 3*/ *es-caspase 7*/ *es-caspase 8* gene expression and distribution during spermiogenesis

The temporal and spatial distributions of *es-caspase 3*/ *es-caspase 7*/ *es-caspase 8* were traced by ISH. In the early spermatids (stage I spermatids), *es-caspase 3* and *es-caspase 8* were in a similar distribution pattern at one pole of the cytoplasm and perinucleus (Figure 2 A1). For *es-caspase 7*, signals were distributed over the whole nucleus and the inner space of the cell membrane (Figure 2 B1, red arrow). No signals were detected in the cytoplasm near the cell nucleus (Figure 2 B1, white arrow). As the PV formation and the nucleus sagging in middle stage spermatids (stage II spermatids), the transcription of *es-caspase 3* and *es-caspase 7* decreased, and that of *caspase 8* enhanced. Part of *es-caspase 3* was concentrated at one pole of the cytoplasm, and the part gathered at the pro-acrosomal cap (Figure 2 A2, red arrow). There was no expression of *es-caspase 3* in nucleus (Figure 2 A2, white arrow). *es-caspase 7* signals still distributed in the nucleus (Figure 2 B2). The enhanced *es-caspase 8* was mainly scattered around the nucleus and pro-acrosomal cap (Figure 2 C2). With cells entering into the terminal stage (stage III or mature spermatids), the crescent nucleus further deformed into cup-like shape and the PV matured into a four-layer structure. *es-caspase 3* unfolded weakened expression relative to the previous stages, was chiefly located in the maturing AT, nucleus and a portion of cytoplasm. The transcription of *es-caspase 7* reached a peak in the mature spermatids, distributed in the whole AV and nucleus, especially the AT. *es-caspase 8* signals were enhanced in the late stage, and had similar distribution patterns with *es-caspase 3* which were located in the maturing AT, nucleus and a portion of the cytoplasm (Figure 2 A3 and C3). Group 4 represented the control group. The above changes of gene expression pattern were illustrated in the diagram model in the upper right corner of each panel.

### Different roles played by Es-Caspase 3, Es-Caspase 8 and p53 during spermatogenesis

**Figure 3:**
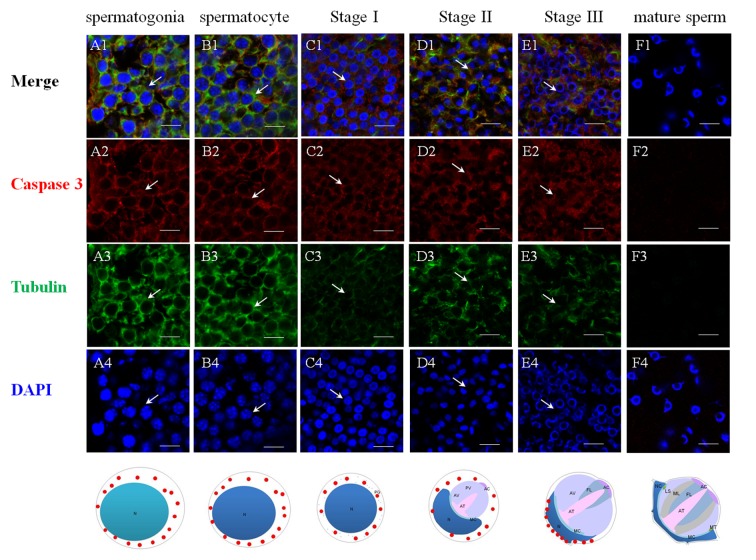
**Immunolocalization of Es-Caspase 3 during spermatogenesis in *E. sinensis***. Red: Es-Caspase 3, Green: Tubulin, Blue: Nucleus. N: nucleus, PG: proacrosomal granule, PV: proacrosomal vesicle, AT: acrosome tube, AC: acrosome cap, AV: acrosome vesicle, FL: fibrous layer, ML: middle layer, LS: lamellar structure, MC: membrane complex, RA: radical arm, NC: nuclear cap, MT: mitochondria. (**A1**-**A4**) spermatogonia, (**B1**-**B4**) spermatocyte, (**C1**-**C4**) stage I spermatid, (**D1**-**D4**) stage II spermatid, (**E1**-**E4**) stage III spermatid, (**F1**-**F4**) mature sperm. Es-Caspase 3 are expressed in the cytoplasm from spermatogonia stage to stage II spermatids. With meiosis progressing, the level gradually decreases. In stage III spermatids, Es-Caspase 3 are concentrated in the cytoplasm between nuclei and cell membrane. No signals are discovered in mature sperm. Bars=20 um.

**Figure 4:**
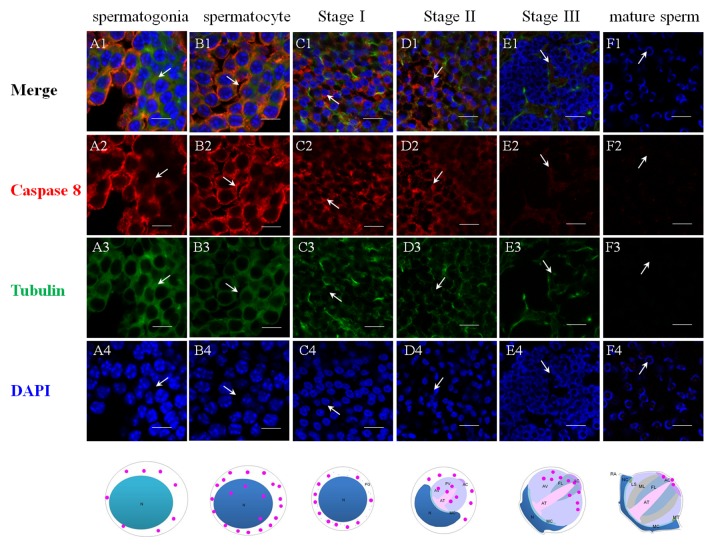
**Immunolocalization of Es-Caspase 8 during spermatogenesis in *E. sinensis***. Red: Es-Caspase 8, Green: Tubulin, Blue: Nucleus. N: nucleus, PG: preacrosomal granule, PV: proacrosomal vesicle, AT: acrosome tube, AC: acrosome cap, AV: acrosome vesicle, FL: fibrous layer, ML: middle layer, LS: lamellar structure, MC: membrane complex, RA: radical arm, NC: nuclear cap, MT: mitochondria. (**A1**-**A4**) spermatogonia, (**B1**-**B4**) spermatocyte, (**C1**-**C4**) stage I spermatid, (**D1**-**D4**) stage II spermatid, (**E1**-**E4**) stage III spermatid, (**F1**-**F4**) mature sperm. Weak Es-Caspase 8 are expressed in the cytoplasm of spermatogonia. In spermatocyte, Es-Caspase 8 are enhanced, distributing in the cytoplasm. Signals transfer in one pole of cytoplasm in the next stage. In stage II and III spermatids, the upper half part of proacrosome present Es-Caspase 8 distribution. In mature sperm, Es-Caspase 8 was distributed in mature AC. Bars=20 um.

**Figure 5:**
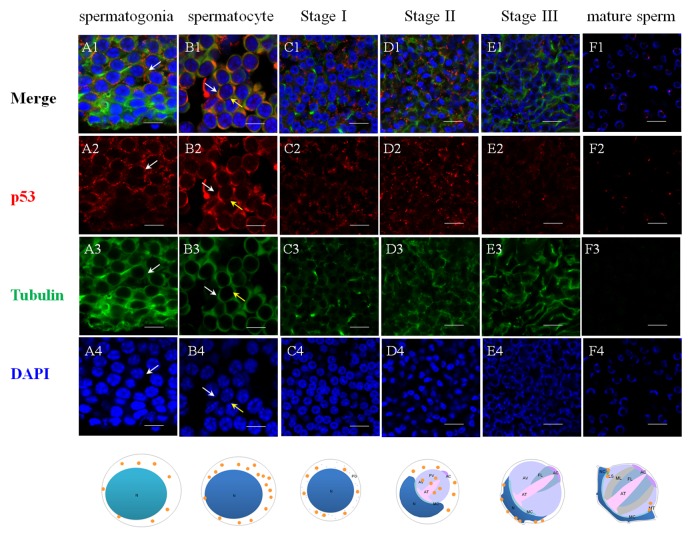
**Immunolocalization of p53 during spermatogenesis in *E. sinensis***. Red: p53, Green: Tubulin, Blue: Nucleus. N: nucleus, PG: proacrosomal granule, PV: proacrosomal vesicle, AT: acrosome tube, AC: acrosome cap, AV: acrosome vesicle, FL: fibrous layer, ML: middle layer, LS: lamellar structure, MC: membrane complex, RA: radical arm, NC: nuclear cap, MT: mitochondria. (**A1**-**A4**) spermatogonia, (**B1**-**B4**) spermatocyte, (**C1**-**C4**) stage I spermatid, (**D1**-**D4**) stage II spermatid, (**E1**-**E4**) stage III spermatid, (**F1**-**F4**) mature sperm. The intensity of p53 in cytoplasm are enhanced from spermatogonia to spermatocyte. In stage II spermatids, signals are found in the proacrosome and partial cytoplasm. In the mature sperm, p53 are found around the rim of the cup-shaped nucleus. Bars=20 um.

The successful preparation of Es-Caspase 3/ Es-Caspase 8/ p53 antibodies provided an experimental basis for the relationship studies between apoptosis factors and spermatogenesis at the protein level. The immunological specificities of all the antibodies were confirmed by WB. In spermatogonia, spermatocytes and stage I spermatids, Es-Caspase 3 presented a spotty distribution evenly in the cytoplasm. In stage II spermatids, Es-Caspase 3 signals reserved in the cytoplasm surrounding the PV. No signals were discovered in the PV. As the nucleus caving in, Es-Caspase 3 shifted into the nucleus and the cytoplasmic space between nucleus and cell membrane (Figure 3 A1). Signals disappeared in mature spermatids (Figure 3 F1). Unlike Es-Caspase 3, Es-Caspase 8 formed its characteristic positioning which indicated its special functions during spermatogenesis. There were scarce Es-Caspase 8 existence in the spermatogonia, and the expression bumped up in cytoplasm when entering into the spermatocyte stage (Figure 4 A1 and B1). The red fluorescence signals (Es-Caspase 8 signal) were at a regular spacing of bead-like order. Fractional parts of the nucleus did express Es-Caspase 8 slightly. Es-Caspase 8 converged to one pole of the cytoplasm, and thereafter saturated the PV in stage II spermatids (Figure 4 D1). The expression level was kept high until stage III spermatids. There was weak detection in the PV. Although very few, Es-Caspase 8 was still expressed in the acrosomal cap in a dotted distribution (Figure 4 E2). The variation of p53 location was depicted by IF results as follows. In the spermatogonia, p53 lined to one side of the cytoplasm in a granulated arrangement. In spermatocytes and stage I spermatids, p53 expression intensified and further assembled into one side of the cytoplasm (Figure 5 B1 and C1). The fluorescence intensity declined and the configuration was punctiform in stage II spermatids. It transferred from the cytoplasm to the PV. The expression was scarce in stage III spermatids and faded away in mature sperm (Figure 5 E1 and F1). The simulative models corresponding to every signal were provided below.

### Different apoptotic responses of Es-Caspase 3, Es-Caspase 8, and p53 induced by Cd^2+^ treatment

**Figure 6:**
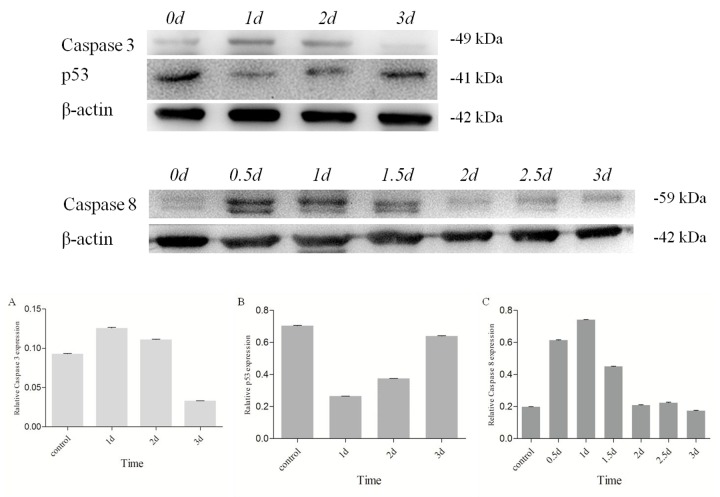
**Changes of Es-Caspase 3/ Es-Caspase 8/ p53 in testis of *E. sinensis* after exposure to 40.28 mg/L Cd^2+^.** Es-Caspase 3 and p53 level were detected by Western blot at 1d, 2d and 3d. Es-Caspase 8 was detected by Western blotting at 0.5d, 1d, 1.5d, 2d. 2.5d and 3d. After 1d induction, Es-Caspase 3 and Es-Caspase 8 increased abruptly. p53 presented the opposite variation. The level of Es-Caspase 3 and Es-Caspase 8 decreased with the increasing of treatment duration while p53 returned to the control level. β-actin was used as a control. All data were resulting from three repeats.

Level of Es-Caspase 3 and p53 in testis treated by Cd^2+^ were measured by western blotting after 1d, 2d, and 3d. The expression of Caspase 8 in testis treated by Cd^2+^ was quantified in 0.5d, 1d, 1.5d, 2d, 2.5d and 3d. Compared to the untreated control group, Es-Caspase 3 and Es-Caspase 8 expression increased while p53 descended sharply after 1d's apoptosis induction (Figure 6). After 1d's induction, the protein expression of Es-Caspase 3 increased by 35%. Es-Caspase 8 changed significantly, nearly four times the normal level. On the contrary, the level of p53 was 40% less than the control group. After induction of 2 days, Es-Caspase 3 kept on increasing with 20% improvement while p53 kept on decreasing with 50% downregulation when comparing with the control group. The level of Es-Caspase 8 decreased to the untreated level in 2 and 3 days. With an interval of two days, the level of Es-Caspase 3 decreased sharply and was 65% less than the control group, p53 continued to drop slightly (Figure 6). After 3d's induction, Es-Caspase 3 and Es-Caspase 8 remained in slashing and achieved a minimum contrast to the control group. In this group, p53 recovered to the level of the control group (Figure 6).

## DISCUSSION

Testicular apoptosis function to sweep away the damaged, defective, superfluous or inadequately supported germ cells [43,44]. Such process could help maintain the structural integrity of the spermatogenic epithelium and keep a healthy environment for testis development. In the present study, three *caspase* family members (*es-caspase 3*/ *es-caspase 7*/ *es-caspase 8*) were identified and characterized in the testis of the Chinese mitten crab *E. sinensis*. In this study, on the base of former research of morphological and biochemical performance in Cd^2+^-induced germ cell apoptosis, we attempt to analyze the functions and potential action mechanisms of Caspases and p53 in testis, particularly during meiotic germ cell division.

### Identification and characterization of Es-Caspases in Chinese mitten crab testis

As proteolytic enzymes, Caspases are implicated in various physiological processes in the crustaceans, such as apoptosis, inflammation, pyroptosis, etc. It has been known that mammalian apoptotic Caspases could be subdivided into initiator Caspases and executioner Caspases based on the existence of N-terminal specific-protein interaction domains [2]. In the light of multiple amino acid sequence alignments and domain architecture predictions, the structural domain composition in Es-Caspase 3 and Es-Caspase 7 are similar to that of some other crabs or shrimp species Caspase 3, which is comprised of the p20 subunit and p10 subunit [16,45]. The difference is that Es-Caspase 3 and Es-Caspase 7 both have another short pro-domain in the N-terminus, with unknown functions. In the Pacific oyster *Crassostrea gigas*, the N-terminal prodomain of Cg-Caspase 3 was found to be indispensable for LPS-binding and caspase activity via ELISA-based LPS binding assay and LPS-Sepharose pull-down assay [46]. We speculated that such pro-domain in Es-Casepase 3 and Es-Casepase 7 may mediate the dimerization of the precursor molecules and the cellular localization [47,48]. In Es-Caspase 8, the conservative DED domain was found between 25 aa and 110 aa. Initiator caspases like Caspase 8 contains a FADD homology domain or DED domain (Caspase-8 and -10), which mediate protein dimerization and/or recruitment into larger complexes to facilitate their activation [2]. As we all know, the active Caspases perform functions after the processing of two successive proteolytic cleavages, depending on the conservative active center. The enzyme active center of Es-Caspase3 was composed of the pentapeptide "NFCRG" located between 326 aa and 330 aa, and Cys328 was predicted to be the key residue. The above active-site conflicted with those in other crustaceans, which is constituted of "QACRG" [15,20]. We explain this with species-specific differences. Similarly, the putative active site of Es-Caspase 7 was the pentapeptide "QACRG". Its actual mechanism of action needs to be disclosed in future studies. Different from other homologous proteins in other species, the active center of Es-Caspase 8 was composed of "QACRG". Phylogenetic trees analysis presented the evolutionary position of Es-Caspase 3, Es-Caspase 7 and Es-Caspase 8 in various species, which further indicating the conservative property of three Caspases (Figure S2 A3-C3). Based on the preliminary analysis, we conclude that both Es-Caspase 3 and Es-Caspase 7 belong to the effector Caspases and Es-Caspase 8 belong to the initiator Caspase. All of them might participate in the regulation of apoptosis in *E. sinensis* testis.

### The broad expression patterns of three *es-caspases* mRNA

As a conserved pathway, apoptosis occurs in various kinds of cells [49]. To confirm the tissue distribution of three *es-caspase* genes in various tissues of Chinese mitten crab, semi-quantitative RT-PCR was conducted. Results show that each *es-caspase* was distributed in a wide range of tissues, including testis, seminiferous duct, seminal vesicle, gill, heart, hepatopancreas and muscle (Figure 1). This result is in accordance with the expression of *caspase-3* mRNA of *Portunus trituberculatus* and *Macrobrachium nipponense* [21,45]. Higher level of *es-caspase 3* was expressed in heart and muscle, which was not aligned with other crabs like *Portunus trituberculatus* [21]. By microarray-based transcriptomics and multiplexed quantitative proteomics analysis and forced expression in Caspases knock-out models, researchers revealed the modulation functions of Caspase 3 in heart’s cellularity and maturation [50]. Different from *es-caspase 3*, a significantly higher level of *es-caspase 7* mRNA was found in hepatopancreas than in other tissues. This may result from the novel functions of caspases in hepatopancreas development. In the male reproductive system, highest *es-caspase 3* and *es-caspase 8* mRNA were found in SV, suggesting their potential roles in the latest stage of spermatogenesis as SV is a storage place for mature sperm. e*s-caspase 7* and *es-caspase 8* exhibited high transcriptional level in the male reproductive system, like testis (Figure 1). The expression patterns of three caspases gave us a hint that they would be responsible for the apoptosis or immune regulation in the testis of *E. sinensis*.

### The underlying functions and onset stage of various apoptotic factors during spermatogenesis

The specific phenomena that characterize spermatogenesis in Chinese mitten crab involves progressively-formed cupped nucleus and the maturing acrosome, which was introduced above. The typical apoptosis characteristics and the enzyme activities detections of several Caspases were described in a number of settings where apoptosis or virus-induced inflammation responses occur [18,30,42,51]. What's unknown is that the location and the extent variations of the three Es-Caspases during spermatogenesis at the transcriptional and translational level, which are determined by stage of germ cell development. The more-refined analyses of *in vivo* patterns of expression of three cell death proteases and another apoptotic factor p53 were accomplished for the first time in the present study by ISH and IF. The changes may reveal the potential apoptotic functions and the relationship with nucleus/ acrosome formation during testis development. In spermatogonia and spermatocytes, the intensity of Es-Caspase 3 and p53 immunostaining was very strong in the cytoplasm, which was in concert with high vulnerability to apoptosis in these two cell types. The intensity of Es-Caspase 8 immunostaining was faint in spermatogonia but became much stronger in spermatocytes. More remarkable, the overall levels of Es-Caspase 3/ Es-Caspase 8/ p53 in early spermatogenic cells were relatively higher compared to other stages during spermiogenesis (Figures 3-5). Actually, some differentiating spermatogonia in mitotic peaks are undergoing apoptosis to ensure tissue homeostasis [52,53]. In normal human testis, apoptosis was mainly observed in primary spermatocytes and spermatids and in a few spermatogonia [54]. It has been reported that exogenous and endogenous signaling molecules participated in the balance of cell proliferation and apoptosis, like the recently discovered vascular endothelial growth factor (VEGFC/VEGFR3), which inhibited spermatogonia apoptosis through Caspase-3/9, PARP and Bcl-2 [55]. As important participants of apoptosis, the precursor proteins Es-Caspase 3 and Es-Caspase 8 in cytoplasm might wait to be cleaved and activated until the apoptosis was launched. In mammals, the role of p53 in testis is to modulate DNA repair and cell apoptosis to control quality and quantity of mature spermatozoa during spermatogenesis [32]. Studies found that p53 homeostasis was maintained by the E3 ubiquitin ligase Cul4 complex in primary spermatocytes to maintain male fertility, as p53 could induce cell cycle arrest to allow DNA meiotic reshuffling and correction of DNA damage [33]. The cell cycle gene p53 also played a critical role in the maintenance of undifferentiated state by inhibiting a mammalian target of the rapamycin complex 1 (mTORC1) in spermatogonia [56]. The successful proliferation and differentiation of spermatogonia and spermatocytes determine the screening of high quality germ cells. In this regards, apart from apoptotic regulation, p53 might participate in the balance of spermatogonia differentiation and self-refreshing. Above all, the three factors might act together to coordinate cell proliferation, differentiation, and apoptosis of spermatogonia and spermatocytes. After the first meiotic division, the dwindling cytoplasmic immunostainings of Es-Caspase 3/ Es-Caspase 8/ p53 were progressively associated with the sagging of the nucleus, with the exception that Es-Caspase 8 and p53 were distributed in the front portion of the acrosomal vesicle in stage II spermatids. As described in the INTRODUCTION, the nucleus deformed to cup-shape and the proacrosomal granules converged into the mature acrosome during spermiogenesis. The difference between Es-Caspase 8, p53 and Es-Caspase 3 may attribute to the different functions in nucleus shape alteration and acrosome forming. The proacrosomal granules may originate from the fusion of Golgi and specialized lysosome-derived vesicles, which means that Es-Caspase 8 and p53 may play an important role in these organelles [57]. In addition, germ cells at the later stages of spermatid morphogenesis will get rid of the unneeded cytoplasmic ingredients, mitochondrial DNA and organelles in a Caspase-dependent process that results in the formation of mature sperm [58]. Here, Es-Caspase 8 and p53 in middle stages might function to assist the formation of the acrosomal vesicle and the maturation of spermatids. In mature sperm, p53 positioned at the edge of the nucleus, while Es-Caspase 8 showed up in the mature acrosome with dotted distribution. As a transcriptional factor, p53 could induce the transcription of Bcl-2 family members and other apoptotic genes [59,60]. In addition, p53 also reacted to DNA damage [61]. Here, the existence of p53 in mature sperm indicated the protective role of p53 at late stage, and p53 could take part in the cell life-or-death choice whatever dangerous factors happened to the cell. However, it was strange that no marked signal of Es-Caspase 3 was found in mature sperm. The complete absence of Es-Caspase 3 might imply either the indispensable role of Es-Caspase 3 or that it must be induced under some circumstances. All in all, the data presented here define for the first time the *in vivo* patterns of expression of Es-Caspase 3, Es-Caspase 7, Es-Caspase 8 and p53 among different stages of male germ cells. The differences in the relative levels and subcellular localization may influence the relative sensitivity or resistance of cells to apoptotic stimuli under some conditions. Beyond that, the successful completion of key events during spermatogenesis depends on these factors, like spermatogonia maintenance, cell differentiation, cytoplasm discarding. It should be noted, however, that the experimental condition or the high detection accuracy of related machines are required before firm conclusions can be reached. Relative activities of Es-Caspase 7 are needed as well with a suitable antibody.

### The effects of Cadmium on the apoptotic responses of Es-Caspases and p53

Heavy metal accumulation was reported to damage different tissues via inducing cell apoptosis in aquatic animals, especially crustaceans. As one of the most toxic environmental and industrial pollutants, the apoptosis-induction functions of Cd^2+^ to various organs in crustaceans have been intensively studied. In the testis of freshwater crab, *Sinopotamon henanense*, Cd^2+^ exposure brought about changes in typical morphological characteristic and oxidative stress, like the fluctuant enzyme activities of superoxide dismutase (SOD), glutathione peroxidase (GPx), catalase (CAT) and variations in malondialdehyde (MDA) [30]. Enzyme measurements of Caspase 3 and Caspase 9 may also indicate the possible participation of mitochondria-dependent apoptosis pathway [30]. In *E. sinensis* and *S. henanense* hepatopancreas, both oxidative damage and Caspase-dependent apoptosis occurred after Cd^2+^-induction [34,51]. In our study, we attempted to reveal how these given apoptotic molecules reacted to Cd^2+^ and how they interacted with each other, finally determining the cell fates all together. From results described in RESULTS **“**Different roles played by Es-Caspase 3, Es-Caspase 8 and p53 during spermatogenesis”, a similar variation trend in protein level was found in Caspase 3 and Caspase 8 post Cd^2+^-adding: suddenly surged at 1d, declined gradually to the level below the control group at 3d. As the apical protease, Caspase 8 could be activated by death ligand and subsequently triggering the downstream executioner Caspases in extrinsic apoptotic pathway [62,63]. The changes of Es-Caspase 8 level indicated the participation of extrinsic apoptotic pathway in Cd^2+^-induced testis (Figure 6). This is different from a previous study which demonstrated the non-function of death receptor pathway [51]. Caspase 3 functions as the effector Caspase in both extrinsic and intrinsic pathways, being in charge of the proteolytic cleavage of many key proteins, such as the DNA repair enzyme nuclear enzyme poly (ADPribose) polymerase (PARP) [64,65]. Surging Es-Caspase 3 levels after Cd^2+^-handling after 1d indicated the increasing demand for apoptotic effectors. As zymogens, Caspase 3 and Caspase 8 are self-spliced or cleaved into two subunits which comprise the active enzyme. In our study, the Es-Caspase 3 and Es-Caspase 8 antibodies accurately detected the pro-enzymes, but not the active protein or subunits. The main reason for the gradual decline in Es-Caspase 3 and Es-Caspase 8 may be the proteolysis of these enzymes (Figure 6). Based on the mechanisms known from mammals, Es-Caspase 3 may be an important substrate of Es-Caspase 8. After 2 or 3 day treatment, the cell may launch massive apoptotic programs to answer oxidative stress or cell death devocators caused by heavy metal element. P53 decreased post 1d treatment, recovered to the level of control group post 3d treatment, which implying the interference of p53 to cell survive or death (Figure 6). The tumor suppressor p53 could assemble multiple stress signals into diverse pro-apoptotic responses [66]. On the one hand, p53 binds and trans-activates target pro-apoptotic genes, like Bax, Apaf-1, Fas/CD95, and others [67–69]. On the other hand, the activation of p53 brings changes in REDOX metabolism, resulting in the increase of ROS and cell apoptosis [42]. The gradual rise in Es-p53 level post Cd^2+^-adding at 1d, 2d and 3d indicated more p53 was needed, which was used for transcriptional control or ROS production. Such phenomenon was in accordance with the oxidative stress elevation during apoptosis in Cd^2+^-handling crabs [30,34,51]. Confusingly, in contrast with the control group, the p53 level decreased abruptly after one day induction (Figure 6). A possible explanation is that the stress responses and the complex regulatory mechanisms in an organism, which need time to coordinate a wide variety of factors.

## CONCLUSION

**Figure 7:**
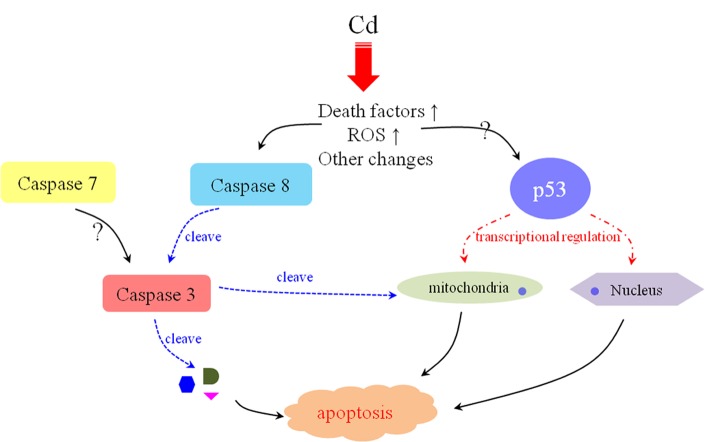
**The possible mechanism of Cd^2+^-induced apoptosis in *E. sinenesis* testis.** The oxidative stress and high-level death factors are triggered by Cd^2+^-induction. On the one hand, these changes initiate Caspase 8-mediated extrinsic pathway. On the other hand, p53 functions to repair DNA damage and participated in the mitochondrial pathway. All of these effects are contributive to the apoptosis of male germ cells after Cd^2+^ treatment in *E. sinenesis*.

In conclusion, three crucial apoptosis-related proteases in *E. sinensis* (Es-Caspase 3/ Es-Caspase 7/ Es-Caspase 8) were isolated and annotated. Three Caspases had a wide distribution and were evolutionarily conserved. In this study, we noted that the variations in Es-Caspase 3/ Es-Caspase 8/ p53 immunostaining patterns were associated with distinct nuclear alterations and acrosome formation. The potential molecular mechanisms of Cd^2+^-induced apoptosis were explored for the first time. We found that the extrinsic pathway involved Es-Caspase 8-Es-Caspase 3 and the intrinsic pathway involved Es-p53 jointly to take effect during spermatogenesis after Cd^2+^-handling. The possible apoptotic signal network triggered by Cd^2+^ is summarized in Figure 7. Figuring out such network in more detail will provide insights into the apoptosis occurrence in crustaceans, and will identify strategies to improve the sperm quality and output quantity in the future. In addition, this work did provide an important foundation for the research of apoptosis in crustaceans, although the molecular mechanisms of action need more detailed investigations.

## MATERIALS AND METHODS

### Animals, sampling, RNA, cDNA and protein preparation

All steps and operations were kept to the strict implementation of the standards from the institutional research ethics committee of the College of life science at Zhejiang University. Eighty healthy Chinese mitten crabs *E. sinensis* were purchased from the crab farm (Caojing Town special aquaculture farm in the Jinshan District near Shanghai, China). Grouping into 16, crabs were acclimated to the uniform filtered and aerated freshwater environment. After anesthetized on ice, different tissues (muscle, gill, heart, hepatopancreas and testis) were collected. Some were stored in -80°C for RNA and protein extraction and the other were fixed in 4% paraformaldehyde (PFA) (pH 7.4) for *in-situ* hybridization (ISH) and immunofluorescence (IF).

50-100 mg crab tissue was homogenized in 1 mL RNAiso Plus (Takara, Japan) for total RNA isolation. Then 0.2 volume of chloroform were added and mixed. After 5 minutes' standing, the supernatant was prepared by centrifugation at 12000 g for 15 min in 4°C. RNA was subsided by mixing with 0.5-1 volume of isopropanol. RNA quality was tested by agarose gel electrophoresis and RNA concentration was tested by a spectrophotometer. 1 µg high-quality RNA was synthesized into first strand cDNA according to the manufacturer's instructions of PrimeScript^TM^ RT Master Mix Kit (TaKaRa, Japan). The synthetic cDNA was stored in -20°C for use.

Total protein was isolated from 50-100 mg crab tissue by RIPA Lysis Buffer (cwbiotech) with protease inhibitor. The concentration was measured by a Bradford Protein Assay Kit (Beyotime, China).

### The amplifications of full-length sequence, bioinformatics analysis

The degenerate primers of three Caspases (Es-Caspase 3/ Es-Caspase 7/ Es-Caspase 8) were designed according to the sequence alignment among homologous sequences of similar species by the CODEHOP software online (https://virology.uvic.ca/virology-ca-tools/j-codehop/). Rapid-amplification of cDNA ends (RACE) was then performed to obtain the 3' and 5' ends of the sequences by 3'-Full RACE Core Set with PrimeScript™ RTase (Takara, Japan) and SMARTer® RACE 5'/3' Kit (Takara, Japan), respectively. All the primers used in our study were listed in Table S1. To obtain the middle fragments, we ran the polymerase chain reaction (PCR) programs as follows: 98°C for 10 sec; 30 cycles of 98°C for 10 sec, 55°C for 10 sec, 72°C for 1 min. To obtain the 3' or 5' ends, we ran the touch-down PCR as follows: 98°C for 10 sec; 14 cycles of 98°C for 10 sec, 62°C (decreased by 0.5°C/ cycle) for 30 s and 72°C for 1 min; then 26 cycles of 98°C for 10 sec, 55°C for 10 sec, 72°C for 1 min; 72°C for 10 min for the final extension. Then the purified PCR products were ligated with pMD19-T vector (Takara, Japan) and sequencing was finished at Biosune Company, Shanghai, China.

Homologous sequences were acquired from the National Center for Biotechnology Information (NCBI) (http://www.ncbi.nlm.nih.gov/). We used Vector NTI Advance 11.5 (Invitrogen) to align Es-Caspase 3/ Es-Caspase 7/ Es-Caspase 8 protein sequences and analyzed the conserved domains. The phylogenetic trees were developed using MEGA 7.0 by the neighbor-joining (NJ) method.

The protein structure models were targeting secondary structure (refering to the site PROSITE (http://prosite.expasy.org/)) and 3D structure prediction (refering to the online server I-TASSER (http://zhanglab.ccmb.med.umich.edu/I-TASSER)).

### Semi-quantitative PCR

The primers for semi-quantitative PCR were designed according to full-length sequences by the software Primer Premier 6 (Table S1). The size of the product was set between 350bp to 600bp. The Chinese mitten crab β-actin (GenBank No. HM053699.1) gene was used as an internal control. PCR program was run as follows: 98°C for 10 sec; 28 cycles of 98°C for 10 sec, 55°C for 10 sec, 72°C for 30 sec. Amplification products were analyzed by agarose gel electrophoresis and grey value analysis was carried out for further comparison by the software Image J. The data were analyzed by one-way analysis of variance (ANOVA) using SPSS 17.0 software. The experiment was repeated at least three times and the P-value should below 0.05.

### *In-situ* hybridization analysis

The frozen sections of testis were prepared at a thickness of 8 um by a sliding microtome. And the riboprobes of *es-caspase 3*/ *es-caspase 7*/ *es-caspase 8* were synthesized by in-vitro transcription, as described below. The cloning, sequencing and confirmation of the 550bp segment were same with full-length cloning (refer to “Animals, sampling, RNA, cDNA and protein preparation”). Then the 550bp segment was inserted into PGEM-T EASY Vector (Promega). SP6 RNA polymerase (Promega) and Digoxigenin-11-UTP (Roche) were used to produce the riboprobes. Finally, the concentration of riboprobes was measured by spectrophotometer. Sections were prefixed by 4% PFA (pH 7.4) for 10 min, and rinsed by 0.1% diethylpyrocarbonate (DEPC)-activated 0.1M phosphate-buffered saline (PBS, pH 7.4) for 10 min at least two times. Tissues were balanced in the equilibrium buffer 5×SSC (sodium chloride 0.75M, sodium citrate 0.075M, pH 7.0) for 15 min, and then infiltrated in the prehybridization buffer (5×SSC, 50% deionized formamide and 40µg/mL denatured salmon sperm DNA) for 2 h in a 55°C water bath. The hybridization buffer was prepared by approximately 300ng/mL of denatured and Digoxigenin (DIG)-labeled riboprobes and the sections were incubated in a 57°C water bath away from light overnight. After rinsing in gradient concentration of SSC (30 min in 2×SSC solution (pH 7.0) at room temperature (RT); 1 h in 2×SSC solution (pH 7.0) at 65°C water bath; and 1 h in 0.1×SSC solution (pH 7.0) at 65°Cwater bath), the tissues were detected via nitroblue tetrazolium chloride and (NBT) (Promega) and 5-bromo-4-chloro-3-indolyl-phosphate (BCIP) (Promega).

### Apoptosis induction by Cadmium treatment

Seventy viable adult crabs (20.0 ± 0.5g) were sampled and divided into six experimental groups and one control group randomly. Dissolve moderate amounts of CdCl_2_ in 20L water to the concentration of 40.28 mg/L (previously determined LC_50_ value [42]). For Cd^2+^-treatment, the crabs were placed in 40.28 mg/L CdCl_2_ solution for 1/6, 1/3, 1/2, 1 of the 72 h, respectively. There were no water-renewing and food-feeding during the period of the experiment. The dead individuals were removed in time. After handling, select energetic individuals randomly and anesthetize them on ice for 10 mins. The crab shell was cut off immediately. Testis from each group was dissected and prepared for protein extraction and Western blot (refer to “Western blot”). Each assay was repeated three times.

### Western blot

The antigens for Es-Caspase 3/ Es-Caspase 8/ p53 were the full-length protein sequences, the protein-specific polypeptides "N'-ERHGSDIDKERLTGT-C'" and the protein-specific polypeptides "N'-PQVIRKKAKTVE-C'", respectively. Full-length sequence of *es-caspase 3* was cloned and linked into the prokaryotic expression vector pET-28a-c(+) Vector (Novagen). The proven *es-caspase 3*-pET-28a-c(+) Vector was sent to the huabio company (Hangzhou) and the rabbit polyclonal antibody for Es-BMP7 was prepared successfully.

Equal amount of protein from different tissues (500µg) was denatured in 5×loading buffer in 100°C for 5-10 min and then loaded in the 12% SDS-PAGE gel for separation. Then the protein was shifted on the methanol-activated polyvinylidene fluoride membrane (PVDF, CWBIO, Beijing, China). 5% skimmed milk-PBST (137mM NaCl, 2.7mM KCl, 10mM Na2HPO4, 2nM KH2PO4, 1mM 20% Tween-20) was used to block the membrane for 2 h at RT. The primary antibodies and secondary antibodies (1:3000) were diluted into 5% skimmed milk-PBST according to the dilution ratio. For Caspase 3 and Caspase 8 primary antibodies, the dilution ratio is 1:2000, for the internal control β-actin (Beyotime, China), the ratio is 1:5000. The membrane was incubated with the primary antibody at 4°C overnight and then washed by PBST for three times, 10 min each. After the binding with secondary antibodies and rinsing, the membrane was exposed with BeyoECL Plus (Beyotime, China) by using a Fluorescent chemiluminescence imaging machine (Tanon, China). The grey value was analyzed by the software Image J and data were further calculated by one-way analysis of variance (ANOVA) through the SPSS 17.0 software.

### Immunofluorescence

The frozen section of testis was permeabilized in 0.3% Triton X-100-PBS (PBST, pH 7.0) for 15 min at RT. The non-specific antigenic interference was blocked by 5% Bovine Serum Albumin (BSA) for 2 h at RT. The primary antibody was diluted with the block buffer (Caspase 3: 1/100 dilution; Caspase 8: 1/100 dilution; p53: 1/100 dilution) and the sections were incubated at 4°C overnight. Then the slices were washed with PBST gently by the shaker for 3 times, 10 min each. Then incubate the slice with fluoresce in isothiocyanate (FITC)-conjugated secondary antibody (1/500 dilution) for 1h at RT. After washing in PBS for 3 times (10 min each), 4,6-diamidino-2-phenylindole (DAPI) was added to stain the nuclei. Zeiss confocal laser scanning microscope (CLSM710) was used to observe the slides via 63×1.4NA immersion oil lenses. The images were exported and analysed by ZEN2009 software (Zeiss) and Photoshop CS5.0 software (Adobe), respectively.

## Supplementary Material

Supplementary File
