# Supplementary material for: Roles of three Es-Caspases during spermatogenesis and Cadmium-induced apoptosis in Eriocheir sinensis
Source: Aging (Albany NY). 2018 May 24;10(5):1146–65. doi: 10.18632/aging.101454 (PMC5990378; doi:10.18632/aging.101454)
Supplement: Supplementary File [file aging-10-101454-s001.pdf]

SUPPLEMENTARY MATERIAL

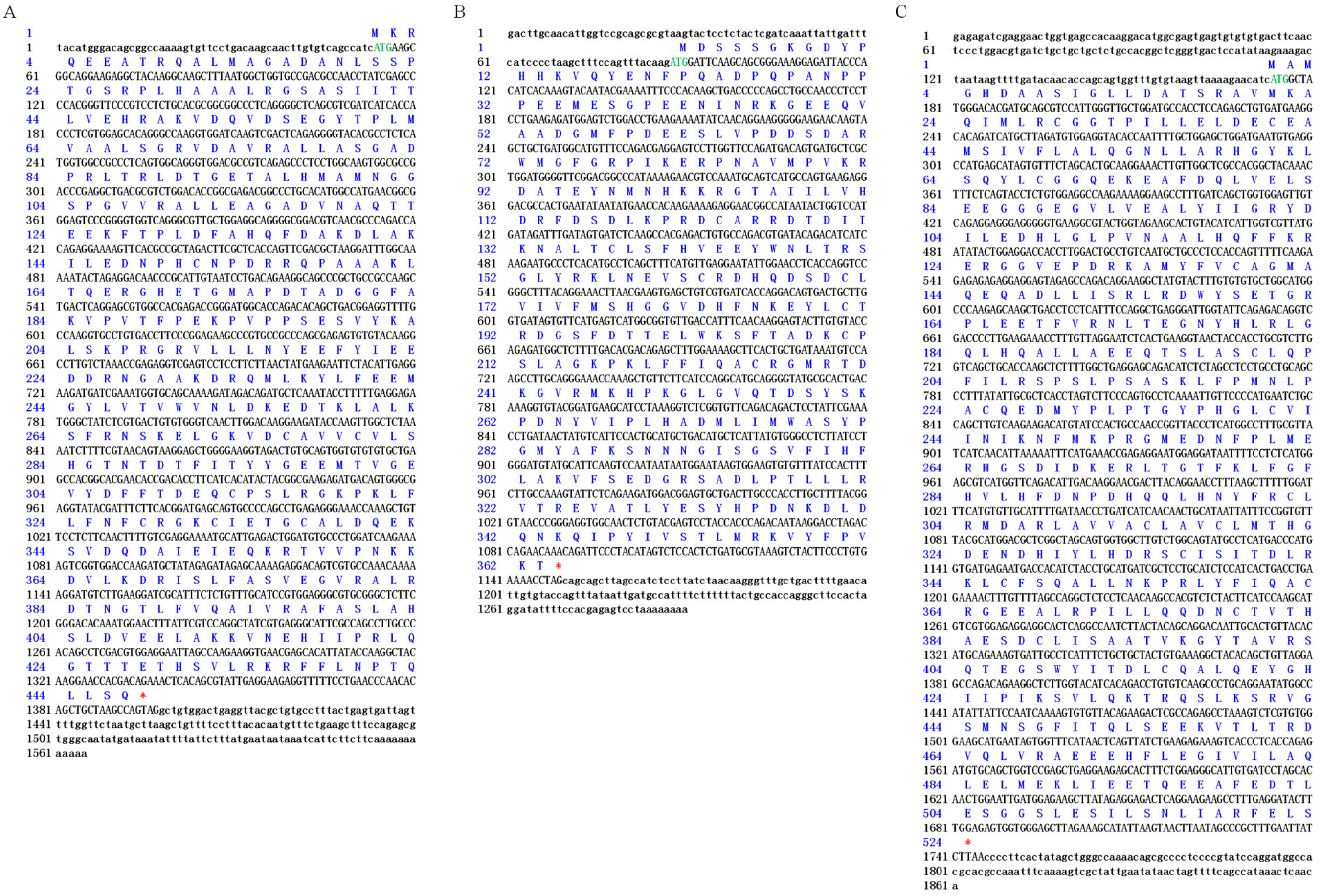

Figure S1. The nucleotide and protein sequences of three Es-Caspases. (**A**) The sequences of Es-Caspase 3. (**B**) The sequences of Es-Caspase 7. (**C**) The sequences of Es-Caspase 8. The "ATG" in green is the start codon, the asterisk (*) indicates the stop codon.

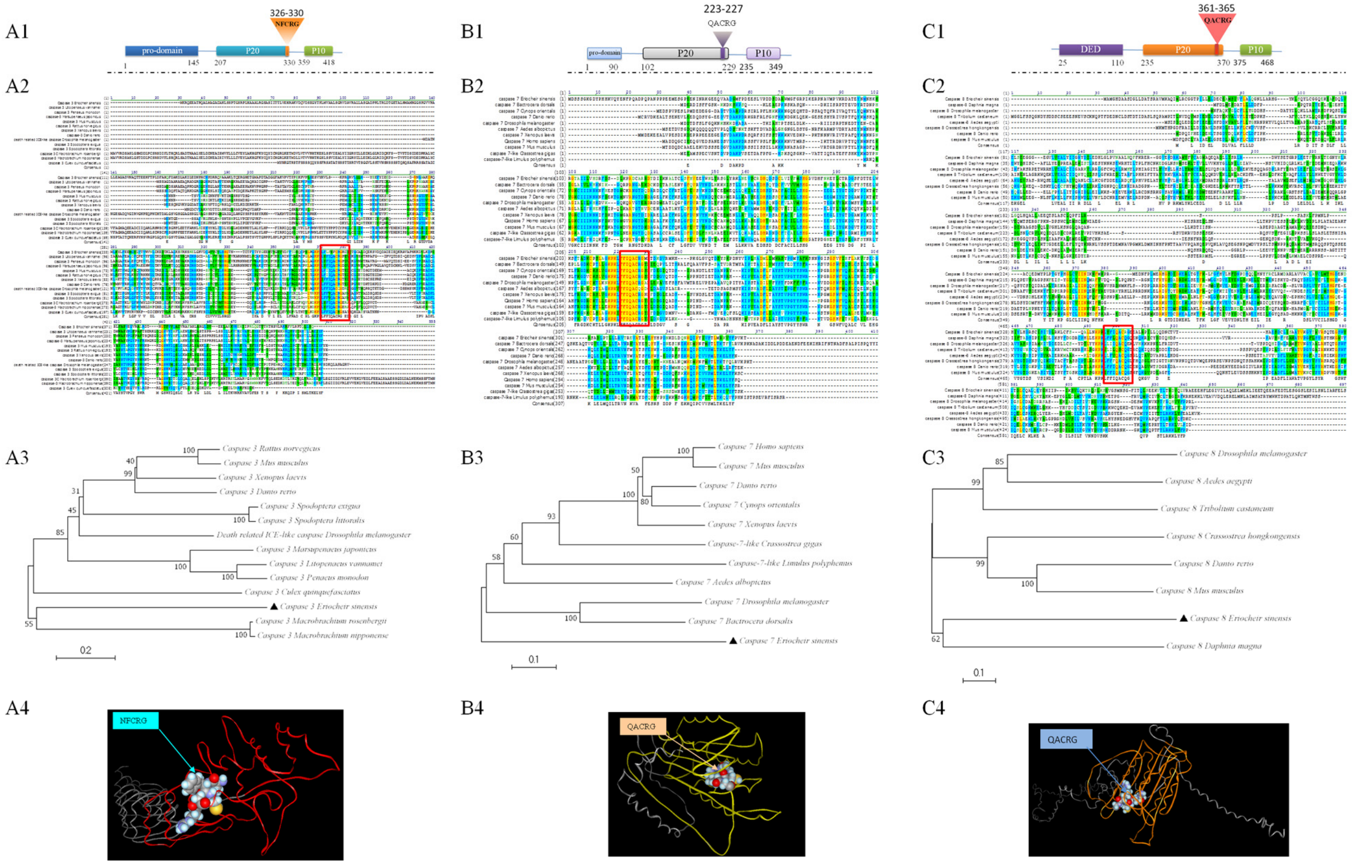

Figure S2. Multiple sequence alignment with selected species and bioinformatics analysis of three Es-Caspases. A1-C1. The prediction of 2D-structure of three Es-Caspases. A2-C2. Sequence alignments of Es-Caspases. Consensus residues are in yellow (identical), blue (conservative) and green (block of similar) colour, the conservative region is in red box. A3-C3. The phylogenetic trees of three Es-Caspases. A4-C4. The predicted 3D-structure of three Es-Caspases.

Table 1. Primers and probes used in this article.

Name	Sequences	application
Caspase 3-mf-F	CTTCDTCCAGTTTTGCCGDGGA	Middle-fragment cloning
Caspase 3-mf-R	CAAGAACTTGCTGCCTCTGAG	
Caspase 7-mf-F	AGTAVGTCCGNACGKCYCC	
Caspase 7-mf-R	CGCHCANGDASTGGATGAA	
Caspase 8-mf-F	CATYCTNTCGCATGGNGA	
Caspase 8-mf-R	NGACYTTCTTYTTCTCRAAN	
Caspase 3-3R-F1	GCTGTTCCTCTTCAACTTTTGTCGA	3'RACE cloning
Caspase 3-3R-F2	AAGTCGGTGGACCAAGATGC	
Caspase 7-3R-F1	GTGCTGACTTGCCCACCTTGCTTTTAC	
Caspase 7-3R-F2	ATGCGTAAAGTCTACTTCCCTGTGA	
Caspase 8-3R-F1	ACAGAAGACTCGCCAGAGCCTAAAG	
Caspase 8-3R-F2	CTTAATAGCCCGCTTTGAATTATCTT	
3'RACE Outer primer	TACCGTCGTTCCACTAGTGATTT	
3'RACE Inner primer	CGCGGATCCTCCACTAGTGATTTCACTATAGG	
Caspase 3-5R-R1	GACAAAAGTTGAAGAGGAACAG	5'RACE cloning
Caspase 3-5R-R2	CTCAGGCTGGGGCACTGCTC	
Caspase 7-5R-R1	TGTACACCTTTGTCAGTGCGCATACC	
Caspase 7-5R-R2	CGCATACCCCTGCATGCCTGGATG	
Caspase 8-5R-R1	CTAACAAAGGTTTCTTCAAGGGGTCG	
Caspase 8-5R-R2	CGACCTGTCTCTGAATACCAATCCCT	
Long primer	TAATACGACTCACTATAGGGCAAGCAGTGGTA TCAACGCAGAGT	
Short primer	CTAATACGACTCACTATAGGGC	
Caspase 3-rt-F	GGGCTATCTCGTGACTGTGTGGGT	Semi-quantitative RT-PCR &ISH probes
Caspase 3-rt-R	GTGAGTTTTTGTCGTGGTTCCTTG	
Caspase 7-rt-F	TTCATGTTGAGGAATCTTGG	
Caspase 7-rt-R	CTTATTGACTGGGTGGTAGG	
Caspase 8-rt-F	AGAGAGGAGGAGTAGAGCCAGAC	
Caspase 8-rt-R	GGTTATCAAAATGCAACACATGA	
β-actin-rt-F	TCGTGCTCGACTCTGGTG	
β-actin-rt-R	GCAGCTCGTAAGACTTCTCC	
Caspase 3-fl-F	ATGAAGCGGCAGGAAGAGGCTACA	Antibody preparation
Caspase 3-fl-R	CTACTGGCTTAGCAGCTGTGTTGGGT	
